# MCMVDRP: a multi-channel multi-view deep learning framework for cancer drug response prediction

**DOI:** 10.1515/jib-2024-0026

**Published:** 2024-09-02

**Authors:** Xiangyu Li, Xiumin Shi, Yuxuan Li, Lu Wang

**Affiliations:** School of Information and Electronics, 47833 Beijing Institute of Technology, Beijing, China; Department of Critical Care Medicine, Renmin Hospital of Wuhan University, Wuhan, Hubei, China

**Keywords:** deep learning, graph convolutional network, convolutional neural network, bidirectional long short-term memory, drug response prediction

## Abstract

Drug therapy remains the primary approach to treating tumours. Variability among cancer patients, including variations in genomic profiles, often results in divergent therapeutic responses to analogous anti-cancer drug treatments within the same cohort of cancer patients. Hence, predicting the drug response by analysing the genomic profile characteristics of individual patients holds significant research importance. With the notable progress in machine learning and deep learning, many effective methods have emerged for predicting drug responses utilizing features from both drugs and cell lines. However, these methods are inadequate in capturing a sufficient number of features inherent to drugs. Consequently, we propose a representational approach for drugs that incorporates three distinct types of features: the molecular graph, the SMILE strings, and the molecular fingerprints. In this study, a novel deep learning model, named MCMVDRP, is introduced for the prediction of cancer drug responses. In our proposed model, an amalgamation of these extracted features is performed, followed by the utilization of fully connected layers to predict the drug response based on the IC50 values. Experimental results demonstrate that the presented model outperforms current state-of-the-art models in performance.

## 1 Introduction

The profound changes in lifestyle, widespread environmental pollution, and the rapid pace of life have contributed to the rise of cancers and malignant tumours as a prevalent disease that poses a serious threat to human life. Currently, drug therapy remains the primary approach for treating these life-threatening diseases. Nevertheless, comparable anti-cancer drug therapies often manifest varying therapeutic outcomes among patients with a resemble type of cancer, attributable to individual variances among cancer patients. Research findings indicate that only a small fraction, approximately 5 % of US cancer patients, had access to genome-targeted treatment classified as efficacious in 2018 [1]. Therefore, there is a significant demand for personalized treatment of cancer patients with tailored drug regimens. It is necessary to elucidate the correlation between drug responses and the genetic profiles of patients with cancer.

Leveraging the Internet advances in medical technology, and the accumulation of medical practitioners has facilitated the creation of numerous extensive datasets concerning drugs and cell lines [2]. Examples of the datasets include the Genomics of Drug Sensitivity in Cancer (GDSC) [3], the Cancer Cell Line Encyclopedia (CCLE) [4], and NCI60 [5]. These resources provide the possibility of computational approaches for predicting drug reactions [6, 7]. Recently, several advanced deep-learning techniques have been developed to capture features from drugs and cell lines, enabling to facilitate the prediction of their interactions. Some methods extract drug features from one-dimensional Simplified Molecular Input Line Entry System (SMILES) [8] strings that represent drug sequences [9], while others extract features from two-dimensional graphs representing the molecular structure of drugs 10], [11], [12], [13. Furthermore, certain approaches combine features extracted from both types of representations [14].

Till now, numerous advanced methods have been developed for predicting drug response, broadly classified into two primary categories: traditional machine learning methods 15], [16], [17 and deep learning strategies 9], [10], [11], [12], [13], [14. However, traditional machine learning methods have two significant drawbacks. Initially, machine learning models are desired to train and test tens of thousands of pairs between drugs and cell lines, so it is unavoidable to take plenty of time due to the limitations of traditional methods. Subsequently, as data volume grows, traditional experimental methods are prone to overfitting due to the increased complexity of the results. So we select deep learning techniques for our experiment.

The recent progress in deep learning has enabled the utilization of a method capable of extracting insights from intricate and high-dimensional datasets, achieving a higher degree of predictive accuracy than employing conventional machine learning techniques. The utilization of deep learning methods could enhance the accuracy of predicting drug responses. Diagrams provide the most intuitive approach for visually representing molecular structures. A molecular graph encapsulates a significant amount of pertinent information about a drug. To extract information from molecular diagrams, the Graph Convolutional Network (GCN) [18], a method that is applied to extract features by progressively aggregating information about the atom and its neighbourhood, is utilized to capture the drug representation [10]. Furthermore, some models employ Graph Attention Networks (GAT) [19] for predicting drug responses [11, 12]. GAT applies attention mechanisms to dynamically adjust the significance of adjacent atoms and their corresponding bond connections. The utilization of the GAT layer facilitates the extraction of information in drugs.

Some authors incorporate Graph Transformer to forecast the drug responses [13]. The Graph Transformer can be viewed as a refinement of GCN. In comparison to GCN, the Graph Transformer demonstrates enhanced capability in representing graph features and capturing intricate relationships between nodes when processing graph data. This advancement holds the potential to enhance the capacity for predicting drug responses. The performance of this approach has been demonstrated to surpass that of traditional GCN.

Given the limitation in obtaining global information from the molecular graph, it is imperative to explore the incorporation of more types of features from drugs. Some methods use one-hot encoding to encode SMILES sequences and integrate them with the molecular graph of drugs to predict responses [14]. The work in this study demonstrates that the use of two types of drug features can improve the accuracy of drug response prediction. Nevertheless, although all these methods yield good results in predicting drug responses, they do not yet fully capture the comprehensive information to represent the intricate characteristics of drugs, indicating that there remains room for improvement. Therefore, our investigation intends to represent the drug from multi-modal perspectives.

Building upon this concept, we introduce a novel model MCMVDRP (Multi-Channel Multi-View deep learning for Drug Response Prediction) to predict drug responses. We utilize three types of drug features: the molecular graph, the drug’s molecular fingerprint, and the sequence of the drug. In our model, three convolutional layers are employed to extract the features of molecular fingerprints [20]. Subsequently, the Graph Transformer, with a combination of the GAT-GCN network is utilized to represent the molecular graph. A three-layer Bidirectional Long Short-Term Memory (Bi-LSTM) block is applied to extract characteristics from the cell line. A homologous structure is also employed to encode features from the drug’s SMILES strings. Finally, the features from all four channels are integrated to forecast the drug responses.

## 2 Methods

**Figure 1:**
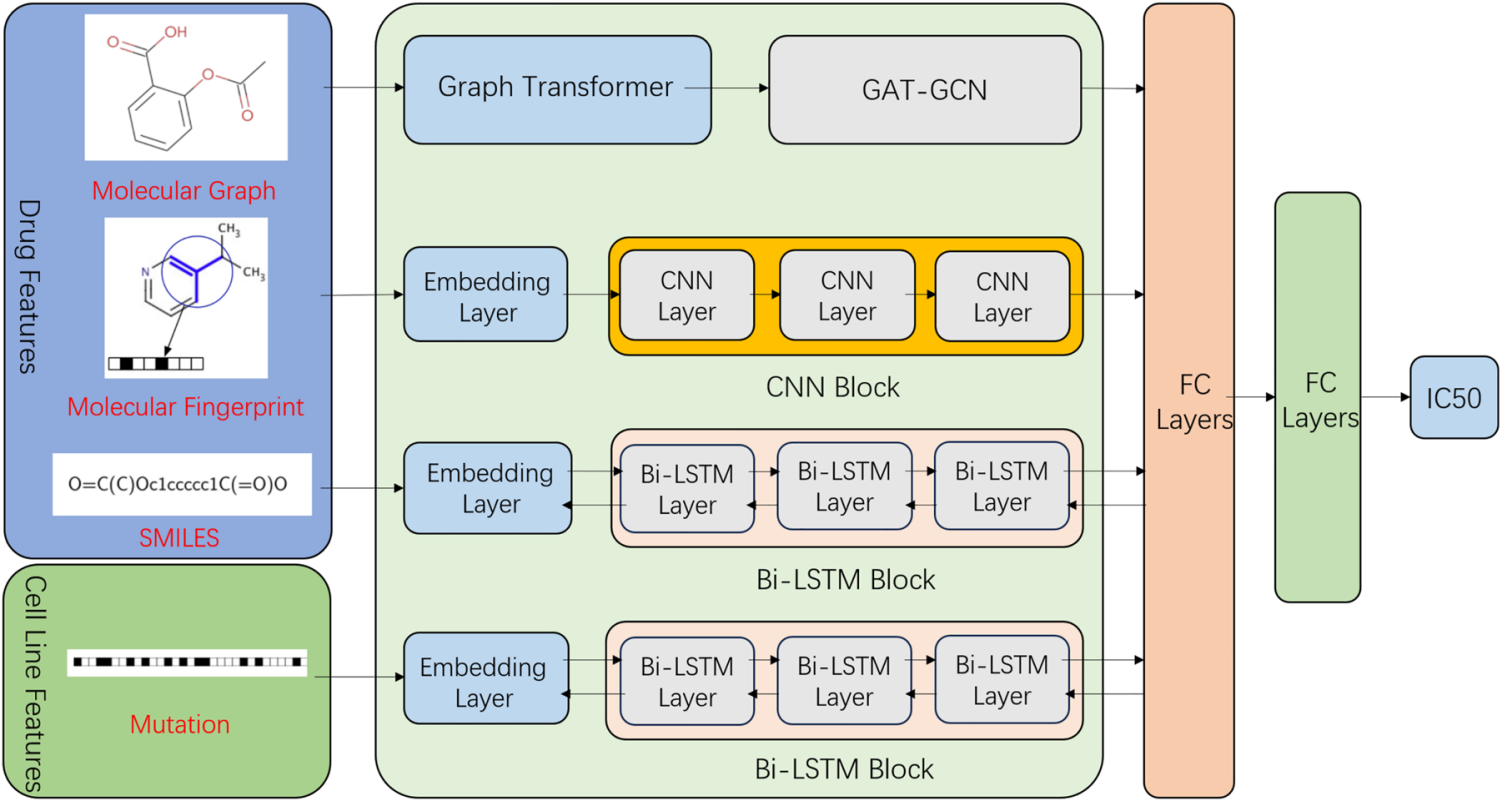
Structure of MCMVDRP.

An overview of the proposed MCMVDRP is depicted in Figure 1. MCMVDRP consists of four channels. Each channel delineates one feature of the drugs and cell lines. The details of these channels to extract features are as follows.

### 2.1 Molecular graph

To transform a SMILE sequence into a molecular graph, the open-source chemical software, RDKit, is employed. This software provides a comprehensive suite of libraries designed for generating molecular graphs that reflect the interactions between the atoms of the drug. In order to characterize the atom features of each node in the graph, it is essential to utilize an atom feature design based on DeepChem [21]. Each node is characterized by five atom features: the atom symbol, the atom degree calculated by the number of bonded neighbors and hydrogen atoms, the atom’s implicit value, whether the atom is part of an aromatic ring, and the total count of hydrogen atoms in the graph. All of these features are encapsulated with a multi-dimensional vector. In our model, a Graph Transformer layer is employed to facilitate the extraction of the features from the molecular graph. Subsequently, a combined GAT-GCN architecture is applied after the Graph Transformer layer. Related research has proved that the hybrid network of GAT [19] and GCN [18] demonstrates superior performance compared to other GCN variants [10].

#### 2.1.1 Graph transformer

In the majority of cases, the molecular structure of a drug may lack interconnections between its constituent nodes, posing a challenge for conventional GCNs to extract adequate information effectively. The Graph Transformer has the capability to enhance the feature representation of a drug when the connections are absent. Formally, a heterogeneous graph *G*, characterized by a set of nodes and edges, includes a tensor *A* representing adjacency and a matrix *X* containing features. The feature matrix of the graph is composed of *N* nodes, each represented by a vector with a dimension of *F*. Furthermore, the adjacency matrix $A_{P}$ of path P is obtained through the multiplication of adjacency matrices in the following manner:

(1) $$A_{P}=A_{t_{l}}\ldots A_{t_{2}}A_{t_{1}}$$

where *A*_*ti*_ is a matric represents the adjacency for the *i*-th edge type of a meta-path. For each provided adjacency matrix, a soft adjacency matrix *Q* is defined as:

(2) $$Q=F\left(A,W_{\phi }\right)=\phi \left(A,\text{softmax}\left(W_{\phi }\right)\right)$$

where *ϕ* represents a convolution layer and *W*_*ϕ*_ *ɛR* ^1×1×*k*^. Subsequently, integrating with a GCN network, the representations of each node are computed as:

(3) Z=σi=1CDi−1AilXW

where || is the join operator and *C* presents the number of channels and *D*_*i*_ is the degree matrix of *A*_*i*_. Furthermore, to enhance prediction accuracy, the Graph Transformer applies Laplacian eigenvectors as the positional encoder. The Laplacian eigenvectors are calculated as:

(4) $$\Delta =I-D^{-\frac{1}{2}}AD^{\frac{1}{2}}=U^{T}\Lambda U$$

where the symbol *U* and Λ denote eigenvectors and eigenvalues, respectively.

#### 2.1.2 GAT and GCN networks

For GAT [19], the calculation of each node feature in each layer is as follows:

(5) $$\alpha \left(Wx_{\dot{i}},Wx_{j}\right)$$

where *W* represents the weight matrix, and *x*_*i*_ means the feature vector of node *i*. The variable *α* denotes the attention coefficient, which means the degree of correlation between node *i* and node *j*.

Subsequently, the attention coefficients are computed as follows:

(6) $$\sigma \left(\underset{j\in N\left(i\right)}{\sum }\alpha_{ij}Wx_{j}\right)$$

where *σ* is a non-linear activation function, and *α*_*ij*_ denotes the attention coefficients.

In the context of GCN [18], the GCN layer can be formally defined as:

(7) $${\tilde{D}}^{-\frac{1}{2}}\tilde{A}{\tilde{D}}^{-\frac{1}{2}}XW$$

where *W* represents a trainable parameter matrix, $\tilde{A}$ is the graph matrix representing adjacency augmented with a self-loop, and $\tilde{D}$ is the graph matrix of diagonal degrees.

### 2.2 Molecular fingerprint

Molecular fingerprints [20] can effectively capture the chemical properties of compounds with high accuracy and simplicity. They are essential for the standardization of molecular representation in chemical datasets by transforming the molecular structures into a uniform format, such as bit vectors or numerical values, suitable for computational analysis. In the context of converting molecular fingerprint information from drugs, the GenMACCSKey function from the RDKit library version 2024.03.1 is employed. This function transforms a drug into a 167-dimensional binary vector that contains a comprehensive set of the drug’s unique chemical features. Following the conversion, the fingerprint vector is passed through an embedding layer, which converts the binary vector into a two-dimensional matrix. Subsequently, three 1-D convolution layers are utilized to extract the features from the matrix.

### 2.3 SMILES sequence

SMILES [15] is a linear notation symbol utilized to encode and represent molecular structures and chemical reactions. In comparison to alternative methods of molecular representation, SMILES encoding offers advantages in terms of uniqueness and space-saving. Before extracting features from the sequences, a dictionary-based approach is applied to convert the sequences into numerical vectors. In this method, a dictionary containing all the characters that may potentially appear in the SMILES strings and their corresponding numbers. With the dictionary, a SMILE string is converted into a numerical vector with a dimension of 100. Subsequently, an embedding layer is employed to transform the features into a two-dimensional matrix. Following this transformation, three Bi-LSTM layers are integrated to capture features from the matrix.

### 2.4 Cell line

Based on the identification of genomic aberrations in a cell line, a binary vector is employed to transform the cell line into a 735-dimensional vector. Consistent with the aforementioned blocks, an embedding layer is initially applied to embed the characteristics extracted from the cell line vectors. Following the embedding process, a matrix that encapsulates the features of the cell line is generated. Subsequently, A Bi-LSTM block with three layers is implemented to capture the feature.

After extracting features from all channels, the data from each channel will be consolidated and processed through two Fully Connected (FC) layers. This step aims to integrate the outcomes of the convolutional and pooled layers, while also effectively mitigating overfitting. The initial fully connected layer had a 512-dimensional input and a 1,024-dimensional output, while the subsequent layer had dimensions of 1,024 and 128, respectively.

## 3 Experiments and results

### 3.1 Datasets

Extensive databases dedicated to drug sensitivity screening include a comprehensive collection of data and information on drug responses to anti-cancer drugs across cell lines. GDSC [3] and CCLE [4] are typical of them. In our experiment, we have selected GDSC, which contains 250 drugs tested across 1,074 cell lines, to evaluate our proposed model. The data contains information on gene expression, indicating the RNA molecules synthesized through the transcription of DNA, ultimately resulting in the translation of proteins within the cell. Therefore, the degree of gene expression signifies the intensity of activity of a particular gene at a specific stage (whether it is a disease state or normal) within a cell. The aim of drug response prediction is to identify the precise drug concentrations needed to effectively inhibit the proliferation of cell lines. Consequently, we have opted to utilize IC50 values that are standardized on a scale ranging from 0 to 1 as the measure for predicting the response between drugs and cell lines.

### 3.2 Baselines

GraphDRP [10]: GraphDRP employs a GCN block to extract the characteristics from the molecular graph of the drug, while also utilizing a CNN to encode the cell line features. The resulting feature vectors of the drugs and cell lines are integrated and inputted into the fully connected layers to predict the drug responses.

GraTransDRP [13]: GraTransDRP utilizes the Graph Transformer layer in conjunction with CNN to represent the features of the molecular graphs and cell lines, respectively. Subsequently, the vectors from these channels are combined, and a fully connected block is utilized for the ultimate prediction.

DGSDRP [14]: DGSDRP combines GAT and GCN to extract molecular graph features. 1D-CNNs are used to encode SMILES representation. The Bi-LSTM layers are employed to capture the features of the cell lines. Finally, the FC layers integrate all the processed data to forecast the drug responses.

The hyper-parameter employed in our model remains the identical configuration outlined in the papers.

### 3.3 Experimental design

In our study, we utilize PyTorch version 2.1.2 for the experiment. The Adam optimizer with a learning rate set at 0.0005 was chosen. To enhance the generalization capabilities of our neural networks, we opted for a training epoch of 300 and a batch size of 256. A dropout layer with a rate of 0.2 was added after each Fully Connected layer to mitigate the risk of overfitting. Notably, the dropout rate for the final layer following the output layer was set at 0.5. The GPU chosen for the training of our model was the Nvidia RTX 4070, with a memory capacity of 12 GB. In the CNN layers of our model, the number of filters increased with each subsequent layer. The initial CNN layer was equipped with 32 filters, the following layer had 64 filters, and the final CNN layer had twice the number of filters as the previous layer. As for the Bi-LSTM layer, the hidden state dimension for the layer was set at 128. Our experiment was conducted using the GDSC dataset. Before splitting the data, we randomized the pairs to mitigate overfitting. Subsequently, 80 % of the dataset was assigned to the training set, 10 % to the validation set, and the remaining 10 % to the testing set. The validation set was partitioned to fine-tune the model, while the testing set was employed to assess the efficacy of the model.

### 3.4 Performance evaluation

In our experiments, we employ two metrics to assess the efficacy of our models: Root Mean Squared Error (RMSE) and Pearson correlation coefficient (CCp).

The Root Mean Square Error is a commonly utilized metric for quantifying the disparity between actual and predicted values. The RMSE is computed as follows:

(8) $$\text{RMSE}=\sqrt{\frac{1}{n}\overset{n}{\underset{i=1}{\sum }}{\left(o_{i}-y_{i}\right)}^{2}}$$

where *n* represents the number of samples, *o*_*i*_ denotes the predicted values, and *y*_*i*_ signifies the actual value of the *i*-th sample.

The Pearson Correlation Coefficient is employed to quantify the relationship between actual values and predicted values. CCp is defined as:

(9) $$\text{C}{\text{C}}_{\text{P}}=\frac{\overset{n}{\underset{i=1}{\sum }}{\left(o_{i}-y_{i}\right)}^{2}}{\sigma_{o}\sigma_{y}}$$

where *σ*_*o*_ and *σ*_*y*_ represent the standard deviations of the predicted values and the actual value, respectively. And *o*_*i*_ represents the predicted value and *y*_*i*_ represents the actual value of the *i*-th sample.

Generally, a lower RMSE and a higher CCp indicate a more effective model.

### 3.5 Experimental results

#### 3.5.1 Performance comparison

To verify the performance of the model we proposed and to compare it with the current state-of-the-art models, we conducted training on the GDSC dataset. The predictive performance was assessed utilizing the CCp and the RMSE metrics. Table 1 displays the comparative performance analysis of our model compared to three other models:

**Table 1:** Prediction evaluation of the proposed model and baselines on the GDSC dataset.

Model	Method		RMSE ↓	CCp ↑
	Drug	Cell line		
GraphDRP	GIN	CNN	0.02425	0.9296
GraphDRP	GAT-GCN	CNN	0.02378	0.9325
GraTransDRP	Transformer	CNN	0.02390	0.9317
DGSDRP	GAT-GCN + CNN	CNN + Bi-LSTM	0.02453	0.9279
MCMVDRP	Transformer + CNN + Bi-LSTM	Bi-LSTM	**0.02334**	**0.9351**

“↓” means the smaller the value, the better. “↑” means the larger the value, the better. For clarity, the best results and the second-best results are highlighted in bold and underlined for each metric, respectively.

The results in Table 1 illustrate that the MCMVDRP model, which utilized three drug features, exhibits superior performance in the prediction of drug response. The superior performance is evidenced by a low RMSE of 0.02334 and a high CCp of 0.9351 values. When compared to the best-performed baseline model, which achieved an RMSE of 0.02378 and a CCp of 0.9325, our proposed model has demonstrated improvements of 1.88 % and 0.278 % in these metrics, respectively. These results indicate that the performance of MCMVDRP outperforms all of the baseline models. The integration of SMILE strings, molecular graphs, and molecular fingerprints potentially offers a more comprehensive representation of valuable features in molecular analysis.

#### 3.5.2 Blind experiment

Considering the utilization of the extensively randomized GDSC dataset in our experiment, it is anticipated that a drug included in the training set will also be found in the validation or testing set. The inclusion of all drugs in the datasets may potentially compromise the credibility of the experiment utilizing mixed data. Therefore, it is imperative to carry out a blind experiment to train the model and assess its efficacy in predicting drug responses for new drugs or cell lines that were not included in the sessions of training. The performance of them is in Table 2.

**Table 2:** Prediction evaluation of the proposed model and baselines with drug blind and cell line data on the GDSC dataset.

Model	Drug blind experiment		Cell line blind experiment	
	RMSE ↓	CCp ↑	RMSE ↓	CCp ↑
GraphDRP_GIN	0.05232	0.4179	0.03665	0.8369
GraphDRP_GAT-GCN	0.04877	0.5272	0.03600	0.8416
GraTransDRP	0.05285	0.5117	0.06636	0.2715
DGSDRP	0.04850	0.5081	0.03408	0.8585
MCMVDRP	**0.04805**	**0.5617**	**0.03392**	**0.8601**

“↓” means the smaller the value, the better. “↑” means the larger the value, the better. For clarity, the best results and the second-best results are highlighted in bold and underlined for each metric, respectively.

Based on the data presented in Table 2, our proposed model also demonstrates superior performance compared to other baseline models in terms of RMSE and CCp in both the drug-blind experiment and the cell line-blind experiment.

#### 3.5.3 Feature ablation experiment

Ablation experiments were carried out to assess the performance of individual blocks in our model in handling drug-related features. In the ablation experiment, a limited number of channels were utilized to extract the drug features. This process yielded six experiments: three for a single channel and three for dual channels. We evaluated the performance of models that represent drug features through the utilization of either single or dual channels. The performance is presented in Tables 3 and 4.

**Table 3:** Performance evaluation of the proposed model utilizing a single channel of drug representation on the GDSC dataset.

Method	RMSE ↓	CCp ↑
SMILES strings channel only	**0.02450**	**0.9282**
Molecular graph channel only	0.03163	0.8784
Molecular fingerprints channel only	0.03529	0.8440
MCMVDRP (all channels)	0.02334	0.9351

“↓” means the smaller the value, the better. “↑” means the larger the value, the better. For clarity, the best results and the second-best results are highlighted in bold and underlined for each metric, respectively.

**Table 4:** Performance evaluation of the proposed model utilizing two channels of drug representation on the GDSC dataset.

Method	RMSE ↓	CCp ↑
SMILES strings channel + molecular graphs channel	**0.02393**	**0.9316**
SMILES strings channel + molecular fingerprints channel	0.02422	0.9298
Molecular graphs channel + molecular fingerprints channel	0.02445	0.9285
MCMVDRP (all channels)	0.02334	0.9351

“↓” means the smaller the value, the better. “↑” means the larger the value, the better. For clarity, the best results and the second-best results are highlighted in bold and underlined for each metric, respectively.

The outcomes of both two ablation experiments demonstrate that each feature we selected from drugs contributes positively to the efficacy of our proposal model. The utilization of the SMILE strings in a single channel and a combination of SMILES Strings and molecular graphs in two channels allows for a more comprehensive representation of drug characteristics by providing a greater amount of accurate information.

#### 3.5.4 Prediction experiment with different types of cancers

**Figure 2:**
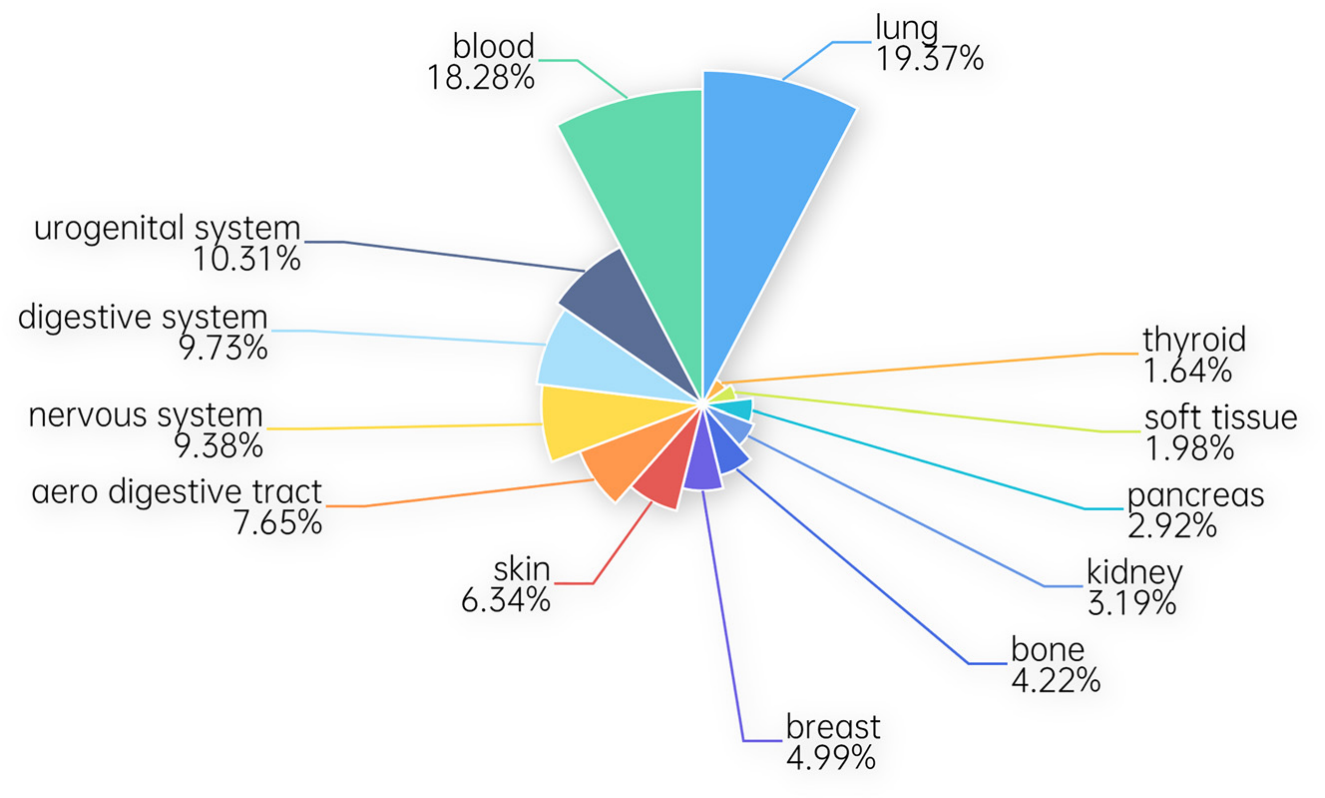
Number of samples of cancer tissues.

In the final phase of our experiment, we conducted our experiment with various types of cancers. Different types of cancer are observed to develop in distinct tissues. The particular cancer-type pairings in the GDSC dataset are outlined in Figure 2.

The illustration clearly shows that the dataset comprises 14 distinct types of tissues with varying numbers of drug-cell line pairs. The accuracy of our model in predicting the responses to a specific cancer type is still unknown. Therefore, it is crucial to conduct experiments with distinct tissue types. In the experiment, the dataset is divided into segments based on various types of tissue. Subsequently, our proposed model is trained to utilize the segmented dataset, and its efficacy is evaluated across a range of cancers. The outcomes of our model are as follows:

The results outlined in Table 5 illustrate that all tissue types within the dataset exhibit excellent performance. Blood is the most effective tissue for the model, displaying RMSE and CCp values of 0.02220 and 0.9363, respectively. Conversely, bone demonstrates the worst performance, characterized by an RMSE of 0.037332 and a CCp of 0.8306.

**Table 5:** Performance evaluation of the proposed model on the GDSC dataset with various types of cancer.

Tissue	RMSE ↓	CCp ↑	Tissue	RMSE ↓	CCp ↑
Aero digestive tract	0.03080	0.8871	Blood	**0.02220**	**0.9363**
Bone	0.03732	0.8306	Breast	0.03301	0.8620
Digestive system	0.02638	0.9122	Kidney	0.03093	0.8706
Lung	0.02571	0.9150	Nervous system	0.02504	0.9149
Pancreas	0.03279	0.8801	Skin	0.03196	0.8812
Soft tissue	0.03314	0.8714	Thyroid	0.02789	0.9155
Urogenital system	0.02492	0.9276			

“↓” means the smaller the value, the better. “↑” means the larger the value, the better. For clarity, the best results and the second-best results are highlighted in bold and underlined for each metric, respectively.

#### 3.5.5 Prediction experiment with the latest dataset

The latest GDSC dataset was released in October 2023, which includes 576,758 drug-cell line pairs involving 624 drugs and 978 cell lines, a significant increase from the 2017 dataset. To access the updated dataset, relevant data can be obtained from the official website at https://www.cancerrxgene.org/. Subsequently, the latest dataset was utilized to evaluate the MCMVDRP model. The comparative performances of the MCMVDRP model using different versions of the GDSC dataset are detailed in Table 6.

**Table 6:** Performance evaluation of the MCMVDRP model on different GDSC datasets from 2017 and 2023.

Model	Dataset version	RMSE	CCp
MCMVDRP	2017	0.02334	0.9351
	2023	0.02217	0.9453

The experimental results indicate that the model achieves good performance on both the 2017 version and the latest dataset.

## 4 Conclusions

In this investigation, we introduce a novel neural network model, termed MCMVDRP, which integrates multiple drug-related channels and is specifically designed for the prediction of drug responses. The multi-view features of drugs used in MCMVDRP include SMILE strings, molecular graphs, and molecular fingerprints. We incorporate three features to represent the drugs in MCMVDRP that distinguish it from other existing models. The model incorporates multiple channels to process different types of data simultaneously, using dedicated feature extraction techniques in each channel. To verify the effectiveness of our model, we conducted a series of experiments. First, the results of the performance comparison experiment show that MCMVDRP achieves significant performance on RMSE and CCp over the state-of-the-art baselines. Second, two blind experiments were performed. Our model also demonstrates superior performance in predicting the responses of both novel drugs and unseen cell lines. After confirming the effectiveness of our proposed model in predicting drug response, we proceed with ablation experiments. The results of the ablation experiments indicate that each of the selected drug features contributes significantly to the predictive accuracy of our model. Finally, our model was evaluated using data from various types of cancer. It is significant to note that evaluation results demonstrate highly satisfactory outcomes across all types of tissues examined. In summary, MCMVDRP demonstrates both biological and clinical significance. It can contribute to the selection of appropriate drugs for the treatment of cancers and malignancies.
